# Research on an Automatic Solution Method for Plane Frames Based on Computer Vision

**DOI:** 10.3390/s26041299

**Published:** 2026-02-17

**Authors:** Dejiang Wang, Shuzhe Fan

**Affiliations:** School of Mechanics and Engineering Science, Shanghai University, 99 Shangda Road, Shanghai 200444, China; fanshuzhe@shu.edu.cn

**Keywords:** structural mechanics, plane frame, object detection, YOLO, structural internal force analysis

## Abstract

**Highlights:**

**What are the main findings?**

- Proposed a structured reconstruction method to bridge visual semantics and mechanics, converting image recognition data into precise inputs for the matrix displacement method.
- Established a novel rapid analysis method based on visual perception that integrates deep learning with traditional mechanics to automatically generate internal force diagrams.

**What are the implications of the main findings?**

- This approach automates the workflow from image input to structural solving, significantly reducing analysis time to seconds by avoiding complex manual modeling.
- It provides a new technical path for intelligent structural analysis, proving highly effective for teaching demonstrations and quick engineering estimations.

**Abstract:**

In the internal force analysis of plane frames, traditional mechanics solutions require the cumbersome derivation of equations and complex numerical calculations, a process that is both time-consuming and error-prone. While general-purpose Finite Element Analysis (FEA) software offers rapid and precise calculations, it is limited by tedious modeling pre-processing and a steep learning curve, making it difficult to meet the demand for rapid and intelligent solutions. To address these challenges, this paper proposes a deep learning-based automatic solution method for plane frames, enabling the extraction of structural information from printed plane structural schematics and automatically completing the internal force analysis and visualization. First, images of printed plane frame schematics are captured using a smartphone, followed by image pre-processing steps such as rectification and enhancement. Second, the YOLOv8 algorithm is utilized to detect and recognize the plane frame, obtaining structural information including node coordinates, load parameters, and boundary constraints. Finally, the extracted data is input into a static analysis program based on the Matrix Displacement Method to calculate the internal forces of nodes and elements, and to generate the internal force diagrams of the frame. This workflow was validated using structural mechanics problem sets and the analysis of a double-span portal frame structure. Experimental results demonstrate that the detection accuracy of structural primitives reached 99.1%, and the overall solution accuracy of mechanical problems in the final test set exceeded 90%, providing a more convenient and efficient computational method for the analysis of plane frames.

## 1. Introduction

In the teaching and learning of structural mechanics, the internal force analysis of plane frames constitutes a fundamental and critical component. Students are required to solve extensive exercises to master computational methods such as the Displacement Method and the Force Method, and to comprehend the mechanical behavior of frames under various loading conditions. However, the traditional solution process is cumbersome and time-consuming: students must manually establish structural models, formulate equilibrium and compatibility equations, perform matrix operations, and finally plot internal force diagrams. This process is not only computationally intensive but also prone to errors in intermediate steps, causing students to expend significant energy on tedious numerical calculations rather than focusing on understanding the underlying mechanical principles and structural behavior. Similarly, in engineering practice, structural engineers often require rapid structural verification of frame structures during the preliminary design phase, such as validating structural responses under dead loads and wind loads. Although existing large-scale Finite Element Analysis (FEA) software offers comprehensive functions, utilizing such tools for the quick verification of simple plane frames is often overly complicated, as the modeling input process remains time-consuming. The ability to rapidly extract information and perform structural calculations directly from structural design schematics would significantly enhance work efficiency during the initial design stage.

In recent years, the rapid development of computer vision and artificial intelligence technologies has provided new avenues for addressing these challenges. By capturing images of paper-based printed plane frame schematics using a smartphone, image recognition technology can be employed to automatically interpret the visual data, extract load and geometric parameters, and perform matrix displacement calculations combined with computational structural mechanics methods. This process generates structural internal force diagrams, enabling the rapid derivation of internal forces directly from the plane frame schematics. This approach serves not only as an auxiliary tool for structural mechanics teaching—helping students verify results and improve learning efficiency—but also provides a convenient and rapid verification method for engineering designers.

Currently, deep learning technology has made significant progress in the field of engineering drawing recognition. Researchers domestically and internationally have conducted extensive studies on hand-drawn engineering sketch recognition [1], architectural drawing symbol detection [2], and engineering drawing text extraction [3]. These achievements demonstrate the feasibility of automated analysis and conversion from images to structural models. However, existing studies typically focus on object detection or text recognition, lacking research on the complete workflow from paper-based printed plane frame schematics to internal force diagrams. Regarding structural analysis, the Matrix Displacement Method has been widely adopted due to its completeness, ease of programming, and strong versatility. Computational programming techniques based on this method are mature, with an emergence of numerous large-scale commercial structural analysis software packages [4] and small-scale structural solvers [5], offering diverse choices for mechanics learners and engineers. However, these software tools impose high requirements on the user regarding the modeling process. They not only require manual input of structure and load information to build models but also necessitate familiarity with the software workflow. Furthermore, they do not support generating internal force diagrams directly from photographic analysis of plane frames, which limits their applicability in scenarios requiring immediate feedback in teaching or rapid verification in early-stage engineering.

Therefore, this study aims to develop an automated analysis method for plane frames tailored for structural mechanics problem-solving and rapid engineering verification. Users can capture the schematic of a plane frame using a smartphone. Through computer vision technology, structural information is extracted to realize a streamlined automated workflow from image acquisition to the visualization of mechanical analysis results. This paper is organized into five sections: Section 1 introduces the research background, significance, and current status; Section 2 presents related work, providing a detailed review of computer vision applications in engineering drawing recognition and the automated development of structural analysis; Section 3 details the proposed methodology, including image acquisition, image pre-processing, the YOLOv8-based detection algorithm, the implementation of the static analysis program, and the internal force plotting algorithm; Section 4 validates the accuracy and practicality of the method through structural mechanics exercises and engineering verification cases; and Section 5 concludes the study and outlines future prospects.

## 2. Related Work

### 2.1. Intelligent Recognition of Engineering Images

Engineering drawings and structural schematics serve as essential media for engineers to comprehend engineering structures and for students to master mechanical principles. The automated and intelligent recognition of these documents has long been a research hotspot at the intersection of civil engineering and computer vision. The digital recognition of engineering drawings facilitates various tasks, including the convenient storage and management of paper documents [6], the structured extraction and secondary development of engineering data [7,8], similarity searches for engineering technical drawings [9], and engineering drawing classification [10]. Early research relied on traditional digital image processing techniques, such as the Hough transform, Canny edge detection, morphological operations [11,12,13], and vectorization algorithms to extract geometric features from images [14]. While these methods performed reasonably well on high-quality vector drawings, they often struggled to extract content from non-vector pixel images, especially those containing photographic noise or hand-drawn-style structural mechanics problems.

With the advancement of computer vision technology, particularly the rise of Convolutional Neural Networks (CNNs), the accuracy of object detection algorithms has significantly improved, gradually replacing traditional image detection methods. Deep learning models represented by YOLO and Faster R-CNN have demonstrated superior performance in symbol detection, text recognition, and image classification. Rezvanifar et al. achieved symbol detection in architectural floor plans based on the YOLO architecture. Even in real-world floor plans with occlusion and clutter, their method successfully detected architectural symbols with low intra-class similarity and variable graphical complexity [15]. Approaches based on Faster R-CNN are also popular for engineering symbol detection. Gao et al. utilized the Faster R-CNN architecture to detect tiny component symbols in piping and instrumentation diagrams (P&IDs) of nuclear power plants, a method that can be extended to electromechanical drawings in the engineering field [16]. Theisen et al. proposed a digitization method for chemical process flow diagrams. They employed the Faster R-CNN network to detect symbol units within the diagrams and simultaneously utilized a pixel search algorithm to detect connections between symbols, ultimately achieving the recognition of process flow diagrams [17]. Jamieson et al. employed two detection models, YOLOv7 and Faster R-CNN, to perform symbol detection on 198 construction drawings. Across 655 symbols, the detection accuracies of the YOLOv7 and Faster R-CNN models were 95.8% and 95.6%, respectively. However, in terms of detection speed, the YOLO-based method was faster than the Faster R-CNN-based method, although both significantly reduced the time required for drawing processing [18]. Furthermore, text is a crucial component present in all engineering drawings. In structural schematics, this includes not only geometric lines but also text annotations associated with graphics; thus, single object detection is insufficient, and Optical Character Recognition (OCR) technology must be integrated. Nguyen et al. developed a Faster R-CNN method to detect symbols and text in scanned engineering technical drawings. They first detected text regions from 4630 technical charts and then recognized individual characters within those regions, achieving an average F1-score of 89% and an exact match accuracy for text recognition of 68.5% [19]. Toral et al. used the YOLO model to detect pipeline specifications and connection points, both of which contain text strings. Tesseract was used to recognize the detected text regions, achieving detection and recognition accuracies of 93% and 94%, respectively [20]. Byun et al. proposed an integrated architecture for unified symbol-text detection and recognition, using Sparse R-CNN as the foundation for the detection module. For text recognition, they developed simplified variants based on CNN and Vision Transformer (ViT). This integrated approach allowed for end-to-end learning between modules, yielding symbol-text detection and recognition accuracies of 97.6% and 95.3% [21]. In the realm of image classification, deep learning-based detection methods have found widespread application. Xie et al. proposed a novel Graph Neural Network (GNN) to classify engineering drawings based on manufacturing methods. For information tables within engineering drawings, they utilized CascadeTabNet for recognition; this model includes HRNet for feature extraction and Cascade R-CNN for bounding box extraction, achieving a detection accuracy of 97%. Experiments on a dataset of 1692 engineering equipment drawings showed that the framework effectively classified engineering drawings with an accuracy of 90.78% [22]. Han et al. proposed a GNN-based method to classify continuous lines detected in piping and instrumentation diagrams, developing a Continuous Line Classification Network (ContLineNet). This network classifies nodes in the continuous line connection graph into eight categories. Training and inference on 7371 lines achieved an average precision of 96.97%, a recall rate of 96.78%, and an F1-score of 96.7% [23].

Although the digital parsing of vector drawings is relatively mature, semantic understanding of pixel-based images, such as textbook exercises and hand-drawn sketches, remains challenging. The core issue lies in the fact that visually recognized primitives are not isolated; specific topological connections exist between them. Additionally, primitives such as structural members and loads must be matched with corresponding physical properties. Joffe et al. observed that existing general-purpose detection models struggle to handle non-standardized structural representations in hand-drawn structural sketches. Recognizing the need for a relatively quick method to evaluate design schemes during the early stages of structural design, they proposed a YOLOv5-based framework. By extracting primitive feature information from images to construct structural models, they successfully achieved parameter extraction and finite element analysis for beams in hand-drawn sketches [1]. Addressing the complex connection relationships between primitives, traditional rule-based methods are often inadequate. Carrara et al. aimed to solve the problems of recognition and topological reconstruction of engineering drawing content. They utilized a Graph Neural Network with graph attention layers to achieve line-level semantic segmentation. By constructing an adjacency matrix between primitives, the model was able to learn deep semantic connections between lines and nodes, with an average classification accuracy of drawing elements exceeding 95% [24].

### 2.2. Automated Structural Mechanics Analysis

The automated solution of structural mechanics problems depends not only on the precision of image recognition but, more crucially, on how to transform unstructured visual information into structured mechanical models. Traditional structural analysis software (such as SAP2000 and ANSYS) relies on cumbersome manual modeling and cannot meet the demands for rapid verification and auxiliary teaching. Therefore, research into interactive automated systems that transition from structural sketches or schematics to structural computational models is of significant importance. Early Intelligent Tutoring Systems (ITS), such as ANDES, began attempting to automatically grade mechanics exercises but required students to draw within specific software environments rather than directly recognizing problem images [25]. With the deepening of research, simplified methods for engineering structural modeling based on finite element analysis have gradually emerged. Hutchinson et al. proposed a unified 2D sketch finite element system called 2DSketchFEA. Similar to the teaching aid software “Structural Mechanics Solver,” this system required users to manually extract data from the structure and input it into the system to achieve automated structural analysis; however, this method did not utilize computer vision technology [26]. Murugappan et al. proposed a simplified finite element analysis tool (FEAsy), software that allows users to rapidly and easily transform, simulate, and analyze structures through freehand drawing. However, this tool constructs models by identifying the user’s stroke order and temporal information. The method relies heavily on accompanying hardware (such as a stylus) and specific drawing habits, making it incapable of processing existing textbook exercises or offline static images [27].

Although the aforementioned lightweight tools offer simplification compared to professional software, from a user experience perspective, it would undoubtedly be more convenient if computer vision could accomplish the parsing of information within structural sketches. Cheng et al. proposed an end-to-end structural analysis method utilizing a selective search algorithm combined with a Convolutional Neural Network (CNN) to detect objects in hand-drawn beam images and predict the linear elastic structural response of hand-drawn beams under loads. Simultaneously, finite element analysis was performed based on a two-node, six-degree-of-freedom beam element. Although this method achieved automation, its essence is a data-driven “black box” prediction, lacking a rigorous mechanical derivation process [28]. Joffe et al. adopted a more interpretable step-by-step strategy. They first employed the YOLO model to detect content within hand-drawn sketches, including beams, supports, numerical values, dimension annotations, and loads. Then, combined with the SimpleHTR model to recognize numerical text, they finally utilized a custom Multi-Layer Perceptron (MLP) to associate geometric and physical features, ultimately achieving the automatic solution of statically determinate beam sketches [1].

## 3. Methodology

**Figure 1:**
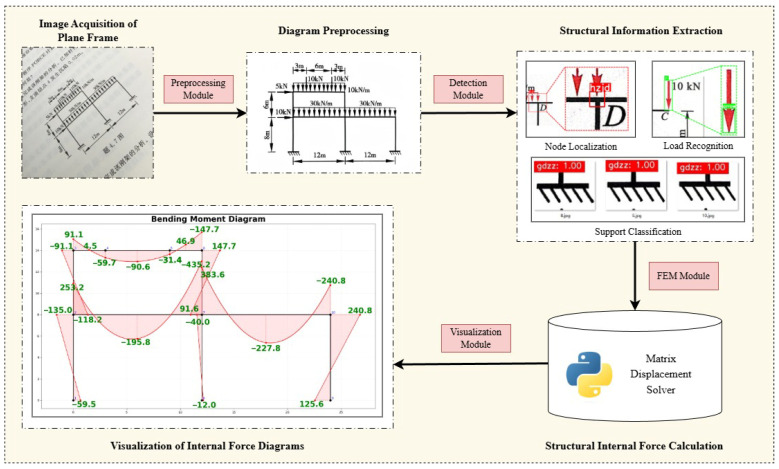
Technical Roadmap.

This study constructs an automated workflow ranging from image acquisition to the output of internal force diagrams. The objective is to realize the capability of “rapidly obtaining mechanical analysis results of paper-based plane frame schematics simply by capturing a photo with a mobile phone.” The overall technical roadmap is organized into five distinct stages:

First, in the image acquisition stage, raw images of plane frame schematics or exercise diagrams are captured using a smartphone under varying lighting conditions, shooting angles, and clarity levels. Second, in the image pre-processing stage, the captured images undergo cropping and rectification, noise reduction, and adaptive binarization to enhance the accuracy of subsequent detection. Third, in the object detection and recognition stage, a frame detection model is trained using the YOLOv8 detection network to extract the geometric and load information of the frame, which is then converted into structured data for subsequent analysis. Fourth, in the finite element calculation stage, the structured data is input into a static analysis program based on the Matrix Displacement Method to generate the global stiffness matrix and solve for internal forces. Finally, in the result visualization stage, the output results from the static analysis program are read to visualize the bending moment, shear force, and axial force diagrams, thereby realizing a complete closed loop of “mobile photography—structural recognition—mechanical solution—internal force diagram output” (Figure 1).

### 3.1. Image Acquisition

Geared towards the application scenario of “rapid analysis of plane frame internal forces via mobile photography,” this study employed mobile phones to collect image samples. The acquisition device employed was a HUAWEI Pura 70 Pro (Huawei Technologies Co., Ltd., Shenzhen, China), featuring a 50-megapixel rear camera, and the raw images were stored in JPEG format with a 24-bit RGB color space. During shooting, it was ensured that the main body of the frame and its annotations were complete and occupied the majority of the view; the resolution of single images was mostly distributed around 3000 × 2000 pixels, providing sufficient pixel density for minute primitives. To approximate real-world usage scenarios, common shooting errors such as image tilt, perspective distortion, and uneven illumination were permitted during the acquisition process, so as to reflect the diversity of practical applications and provide a data foundation for subsequent algorithm robustness verification. The collected frame types include simply supported beams, multi-span beams, portal frames, and multi-story frames. Load types encompass common forms such as concentrated forces, distributed loads, and bending moments.

### 3.2. Image Pre-Processing

Image pre-processing constitutes the first critical component of the frame recognition system. Its objective is to transform mechanics exercise images captured by mobile phones into a format suitable for subsequent object detection and recognition. Raw images acquired via mobile phones often suffer from issues such as perspective distortion, uneven illumination, and noise interference. To enhance the detection precision of the subsequent YOLOv8 model and the accuracy of structural topology reconstruction, pre-processing of the raw images is mandatory. The pre-processing workflow in this section is as follows: First, the Region of Interest (ROI) is extracted through interactive cropping to rectify the geometric distortion of the image. Subsequently, steps including image denoising, adaptive binarization, and morphological processing are performed to obtain clear plane frame diagrams.

#### 3.2.1. Interactive ROI Extraction and Geometric Distortion Rectification

**Figure 2:**
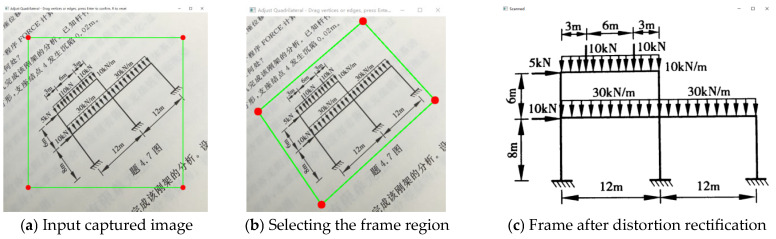
Rectification Workflow Diagram.

Paper-based printed images captured by mobile phones typically contain substantial background information, such as adjacent problems or book edges. This irrelevant information contributes nothing to the solution process and adversely affects feature extraction. To accurately locate the effective region of the frame exercise, this study employs an interactive Region of Interest (ROI) extraction method. This method first generates an initial box on the input image based on default geometric coordinates, as shown in Figure 2a; this initial box operates independently of image features. Users manually delineate the area containing the frame by dragging four control points and four borders on the interface. The system incorporates a linkage mechanism: when a border is dragged, the corner points at both ends move synchronously along the normal vector direction, whereas dragging a corner point individually allows for arbitrary degrees of freedom. The interface frame displays the selected contour in real-time, providing the user with excellent visual feedback. Compared to fully automated edge detection methods, interactive extraction can more robustly handle low contrast, uneven illumination, and complex textured backgrounds, thereby enhancing the robustness of ROI localization (Figure 2).

**Figure 3:**
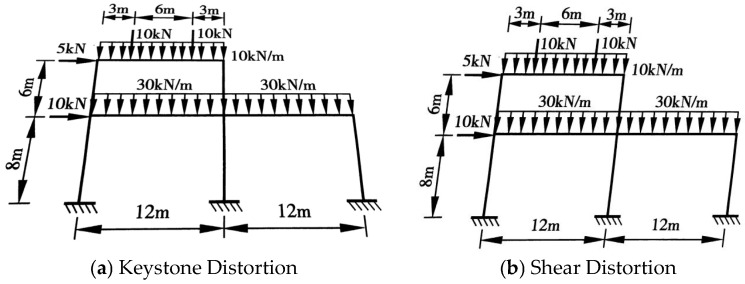
Distortion of Frame Diagram.

Due to the difficulty in maintaining the camera’s optical axis perfectly perpendicular to the paper surface during shooting, the captured image inevitably exhibits projective distortion. Consequently, originally orthogonal rectangular frames appear as trapezoids, parallelograms, or arbitrary convex quadrilaterals in the photograph (Figure 3). This distortion leads to a misalignment between structural members and dimension annotations, resulting in accurate length and angle measurements, thereby compromising the precision of the subsequent geometric analysis. In this study, a perspective transformation method based on the homography matrix is adopted for geometric rectification [29]. Utilizing the principles of projective geometry, this method establishes a mapping relationship between the distorted image plane and the standard orthogonal plane. Let the image coordinates of the four corner points finally determined by the user be $P_{i}=(x_{i},y_{i}),i=1,2,3,4$, and the corresponding corner coordinates in the target rectified image be ${P\prime }_{i}=({x\prime }_{i},{y\prime }_{i})$. The perspective transformation can be expressed by a 3 × 3 homography matrix H:

(1) $$\left[\begin{array}{c} {x\prime }_{i}\\ {y\prime }_{i}\\ 1 \end{array}\right]=H\left[\begin{array}{c} x_{i}\\ y_{i}\\ 1 \end{array}\right],H=\left[\begin{array}{ccc} h_{11} & h_{12} & h_{13}\\ h_{21} & h_{22} & h_{23}\\ h_{31} & h_{32} & h_{33} \end{array}\right]$$

By normalizing with $h_{33}=1$, the eight degrees of freedom (DOFs) of matrix H can be determined. Each pair of corner points corresponds to two linear constraint equations, allowing for the construction and solution of a system of equations. In terms of algorithmic implementation, the width and height of the rectified canvas are first calculated based on the Euclidean distance of the vertices to determine the target point coordinates. The cv2.getPerspectiveTransform function from the OpenCV library is then employed to compute the homography matrix, and the cv2.warpPerspective function is used to execute the perspective transformation [30]. The dimensions of the rectified image are determined according to the following strategy:

(2) $$Width:W=\max\left(||{P\prime }_{2}-{P\prime }_{1}||,||{P\prime }_{3}-{P\prime }_{4}||\right)$$

(3) $$Height:H=\max\left(||{P\prime }_{4}-{P\prime }_{1}||,||{P\prime }_{3}-{P\prime }_{2}||\right)$$

where $||{P\prime }_{j}-{P\prime }_{k}||=\sqrt{{\left({x\prime }_{j}-{x\prime }_{k}\right)}^{2}+{\left({y\prime }_{j}{-y\prime }_{k}\right)}^{2}}$ denotes the Euclidean distance between two points.

#### 3.2.2. Image Denoising and Binarization

Although the image after perspective transformation has achieved geometric rectification of the frame form and removed irrelevant information, further processing is still required to highlight structural features and suppress noise interference. This study employs a combined method of grayscale conversion, Gaussian filtering, adaptive thresholding binarization, and morphological processing to achieve image optimization.

1. Grayscale Conversion and Gaussian Filtering: First, the RGB color image obtained after perspective transformation is converted into a grayscale image using the formula:
  (4) Gray=0.299R+0.587G+0.114B
  To suppress high-frequency noise and smooth details in the image, Gaussian filtering is applied to the grayscale image for denoising. The Gaussian filter is a linear smoothing filter whose convolution kernel weights follow a two-dimensional Gaussian distribution [31]:
  (5) $$G(x,y)=\frac{1}{{2\pi \sigma }^{2}}e^{-\frac{x^{2}+y^{2}}{{2\sigma }^{2}}t}$$
  where σ is the standard deviation, controlling the degree of smoothing. In this section, a 3 × 3 Gaussian kernel is used with a standard deviation σ=0 (automatically calculated by the OpenCV library based on the kernel size), which effectively removes noise while preserving edge information.
2. Adaptive Binarization: To convert the image into a black-and-white binary image to highlight the contours of the frame structure, Otsu’s adaptive thresholding method is adopted. Otsu’s method assumes that the image consists of two classes of pixels: foreground and background. By iterating through possible thresholds in the image, the inter-class variance is calculated for each; a larger inter-class variance indicates a more distinct grayscale difference between the two classes and a better segmentation effect. Therefore, Otsu’s method automatically determines the optimal threshold T* by maximizing the inter-class variance [32]:
  (6) $$T^{*}=\underset{T}{\mathrm{argmax}}\sigma_{b}^{2}(T)$$
  (7) $$\sigma_{b}^{2}=\omega_{0}(\mu_{0}-\mu )+\omega_{1}(\mu_{1}-\mu )$$
  where $\mu_{0}$ and $\mu_{1}$ are the average gray levels of the background and foreground pixels, respectively; $\omega_{0}$ and $\omega_{1}$ are the proportions of background and foreground pixels; and μ is the global average gray level. After determining the optimal threshold T*, the inverted binarization mode (THRESH_BINARY_INV) is employed, setting pixels with values greater than the threshold T* as the white foreground and those less than T* as the black background.
3. Morphological Processing: The binarized image may still contain defects such as isolated noise points and broken lines, necessitating repair through morphological operations. Therefore, a combined strategy of closing and opening operations is adopted [33]:
  Closing Operation: Dilation followed by erosion, used to fill small holes in the image and connect broken lines:
  (8) A•B=(A⊕B)⊖B
  Opening Operation: Erosion followed by dilation, used to remove small isolated noise points:
  (9) A○B=(A⊖B)⊕B
  where A is the input image, B is the structuring element, and ⊕ and ⊖ denote the dilation and erosion operations, respectively. This section employs a 3 × 3 rectangular structuring element, first executing the closing operation to fill lines, and then the opening operation to remove isolated noise. To eliminate the influence of boundary noise, a 2-pixel wide black border is drawn on the image edges after morphological processing. Finally, the processed result is inverted to render the frame structure as black lines against a white background, yielding a clear binarized frame image that provides high-quality input for subsequent object detection.

### 3.3. Object Detection Based on YOLOv8

YOLOv8 (You Only Look Once version 8) is an object detection algorithm released by Ultralytics in 2023, which strikes a favorable balance between detection accuracy and inference speed. The model adopts an anchor-free detection strategy, eliminating the reliance on predefined anchor boxes found in traditional methods and instead directly predicting the object center point and bounding box dimensions. This mechanism reduces the number of hyperparameters and enhances the generalization capability for objects of varying scales, offering significant advantages in handling the substantial scale disparities among primitives in plane frame schematics. Furthermore, YOLOv8 provides five model variants of different scales—n, s, m, l, and x—allowing for a flexible trade-off between accuracy and speed according to practical requirements.

The YOLOv8 network architecture comprises three primary components: the Backbone, the Neck, and the Head. Specifically, the Backbone is built upon the CSPDarknet structure, integrating the C2f module to enhance feature transmission efficiency and improve gradient flow capability. The Neck utilizes the PAN-FPN structure for multi-scale feature fusion, enabling the model to effectively resolve target information at various resolutions while retaining a lightweight profile. The Head component employs a Decoupled Head design, which separates classification and regression tasks, thereby improving training stability and detection accuracy [34]. This study utilizes the YOLOv8-m variant for training.

#### 3.3.1. Dataset Construction

This study constructed a dataset comprising a total of 7030 images, derived entirely from printed plane frame structure diagrams found in textbooks, problem sets, and professional examination papers. This aimed to ensure the standardization of primitive features to satisfy the rigorous requirements of mechanical calculations.

The target types contained within plane frame structural schematics are diverse and exhibit significant morphological variations, including nodal regions (containing nodes), loads (containing arrows), and dimension annotations. Given the orders-of-magnitude differences in pixel dimensions among these targets and the inherent class imbalance between categories, directly employing a single model for all-class detection is prone to causing the features of small targets to be overwhelmed. To address the complexity of information extraction from plane frame schematics and the requirements of the subsequent computational workflow, this study adopts a staged multi-model detection strategy. Seven targeted datasets were constructed to train independent detection models respectively.

The specific dataset division and sample distribution are presented in Table 1. All datasets were manually cleaned and annotated, and partitioned into training, validation, and test sets according to an 8:1:1 ratio.

#### 3.3.2. Model Training and Parameter Configuration

In this study, the YOLOv8 framework was employed to independently train models for each of the seven datasets. All models utilized a transfer learning approach, performing fine-tuning based on the pre-trained weights of YOLOv8-m. The training environment was configured as follows: the processor was an Intel^®^ Core^TM^ i7-8700K CPU @ 3.70 GHz, the GPU was an NVIDIA GeForce RTX 3080, and the memory (RAM) was 48 GB. The software environment included Python 3.9 running on the Windows 10 operating system. The specific parameters for each model during the training phase are presented in Table 2.

#### 3.3.3. Detection Workflow and Metric Analysis

To accurately extract structural information from complex frame diagrams, this study designed a cascade detection workflow based on a “global localization–local subdivision” strategy. The specific steps are as follows:

1. Global Preliminary Screening: The raw frame images are uniformly rescaled to a resolution of 1024 × 1024 and input into four detection models: “Node_area”, “Dimension”, “Load”, and “Value”. This step aims to rapidly locate the spatial coordinates of four types of primitives: node regions, dimension symbols, load regions, and numerical value regions.
2. Region Cropping: The detected node regions and load regions are cropped using image processing algorithms and rescaled to a resolution of 640 × 640 for input into subsequent detection models. Simultaneously, the support node segments within the node regions are filtered out and rescaled to a resolution of 320 × 320 for input into the subsequent classification model.
3. Detailed Fine-grained Classification: The cropped support node images are input into the “Support_Cls” model to determine the support type and mechanical boundary conditions. The node region images are input into the “Node” model to obtain precise node coordinates. The load region images are input into the “Load Vector” model to identify arrows for determining the direction of the loads.

**Figure 4:**
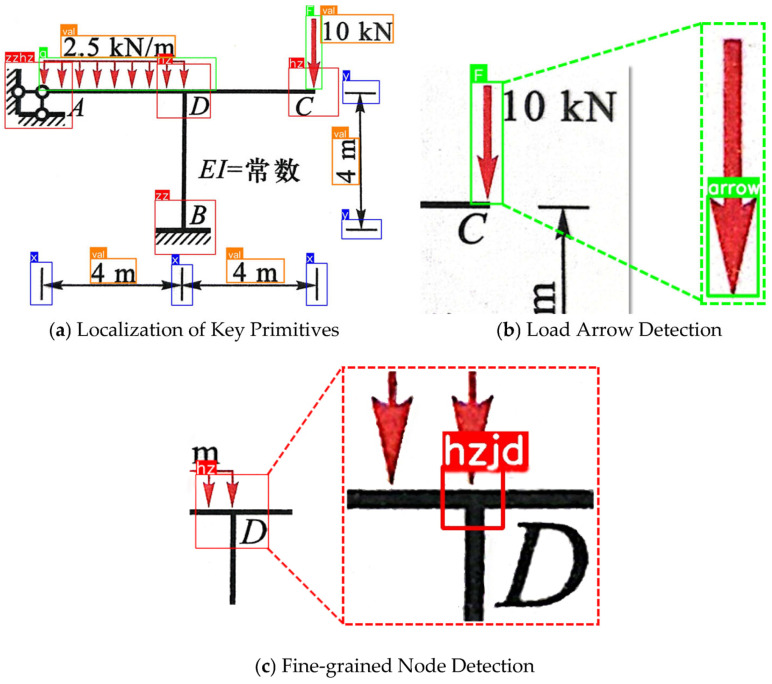
Detection Results of Structural Components. (**c**) The label “hzjd” in image represents the “load node” category in the fine-grained node dataset.

Through the aforementioned workflow (Figure 4), this method effectively extracts valid information from plane frames while avoiding the high missed detection rate associated with directly detecting minute details within large-scale global images.

To quantitatively evaluate model performance, this study adopts Precision (P), Recall (R), and Mean Average Precision (mAP@0.5) as evaluation metrics (Table 3). Specifically, mAP@0.5 represents the mean average precision when the Intersection over Union (IoU) threshold is set to 0.5. Furthermore, for the “Support_Cls” classification model, Top-1 Accuracy is utilized as the evaluation metric.

As indicated by the data in Table 3, the multi-model detection framework proposed in this study demonstrates significant superiority across all metrics. The analysis is as follows:

1. High Precision: With the exception of the support classification model, the mAP@0.5 for all detection models exceeded 0.98. This is primarily attributed to two factors: First, the processed plane frame images exhibit distinct features and clean backgrounds. Second, the multiple ROI cropping method employed in this study effectively eliminated irrelevant features, enabling subsequent models to focus on local feature extraction.
2. High Recall: The recall rates for all detection models were above 0.99. Such an exceptionally high recall rate ensures the reliability of structural calculations, as in the task of extracting structural mechanics parameters, the omission of data is far more critical than false detections.
3. Accurate Classification: The “Support_Cls” model achieved a classification accuracy of 97.2%, ensuring the establishment of correct boundary conditions for the frame. Meanwhile, the “Load Vector” model achieved both P = 1 and R = 1, perfectly resolving the issue of load direction determination.
4. Inference Efficiency: In terms of inference efficiency, the inference time for macro-models processing large-resolution inputs was controlled at approximately 28 ms, whereas models processing local small images (such as “Support_Cls”) required only 4 ms. The inference speed satisfies the requirements for real-time processing.

### 3.4. Frame Data Recognition

The core of this study lies in transforming the visually recognized plane frame diagrams into a structured data file that can be invoked by the subsequent static analysis program. This file comprises the following five groups of data:

1. Basic Topology and Material Parameters: Total number of frame nodes, total number of elements, number of loaded nodes, number of support nodes, and material elastic modulus;
2. Nodal Geometric Information: Node ID, X-coordinate, and Y-coordinate;
3. Element Connectivity and Sectional Properties: Element ID, node IDs at both ends of the element, cross-sectional area, and moment of inertia;
4. Nodal Load Vectors: Node ID, load in the X-direction, load in the Y-direction, and bending moment;
5. Boundary Conditions: Support node ID, indicator information regarding the displacement status of the node in the X, Y, and rotational directions, and the known displacement values in these three directions.

This section is primarily organized into three parts: node localization and topological reconstruction, load recognition and numerical association, and support classification and boundary extraction, covering the extraction of all requisite data.

#### 3.4.1. Node Localization and Topological Reconstruction

According to the discretization principles of the Matrix Displacement Method, the first step involves structural identification of the plane frame, which includes the determination of nodes and elements. Typically, nodes are defined as intersection points of members or free endpoints, while elements are straight bars connecting two nodes. To facilitate structural discretization, two special cases exist for node identification: when a concentrated load exists at the mid-span of an element, an additional node is added at the point of load application; when an element is subjected to a distributed load, the start and end points of the distributed load are treated as nodes.

**Figure 5:**
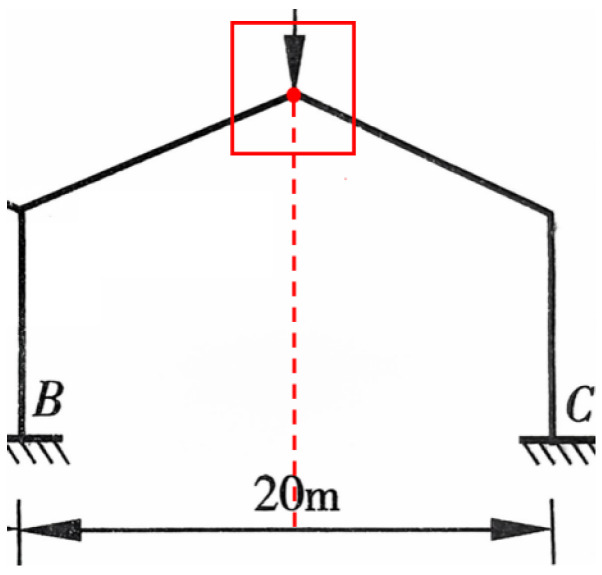
Node without Corresponding Dimension in the X-direction.

This section proposes a node geometric parsing method that combines visual detection with dimensional constraints. Through a “Node Detection—Dimension Detection—Coordinate Constraint” workflow, this method maps the image pixel space to the engineering physical space:

1. Two-Stage Node Detection. A coarse-to-fine detection strategy is adopted. First, a regional object detection model is utilized to locate node regions (ROI) within the overall frame. Subsequently, a fine-grained detection model is employed to precisely locate the node centers within the cropped regions, thereby eliminating background noise interference and obtaining the pixel coordinate set N of the nodes:
  (10) $$N=\{(id_{i},x_{i},y_{i})\}$$
  where $id_{i}$ is the node ID; $x_{i}$ and $y_{i}$ are the node pixel coordinates.
2. Semantic Parsing of Dimensions. To establish the mapping between pixels and physical lengths, the study obtains positioning information by detecting the endpoints of dimension lines and acquires annotation values through Optical Character Recognition (OCR), constructing a dimension dataset D:
  (11) $$D=\{(x_{s},x_{e},y_{s},y_{e},d,\lambda )\}$$
  where $\left(x_{s},x_{e}\right)$ and ${(y}_{s},y_{e})$ are the pixel coordinates of the start and end points of the dimension; d is the dimension value; and λ is the direction indicator factor (0 for horizontal dimensions, 1 for vertical dimensions).
3. Node-Dimension Spatial Association. Since a single dimension annotation in an image often spatially corresponds to multiple nodes, association via coordinate projection is required. Therefore, the dimension endpoints are matched with structural nodes in the horizontal and vertical directions, respectively. A tolerance threshold δ is set. For horizontal dimensions (λ=0) nodes in set N satisfying $\left|x_{i}-x_{s}\right|<\delta$ are sought as start-point associated nodes; end-point associated nodes are matched similarly. Vertical dimensions follow the same logic. A node-dimension association array R is constructed:
  (12) $$R=\left[x_{s},x_{e},y_{s},y_{e},d,\lambda ,id_{x,s},id_{x,e},id_{y,s},id_{y,e}\right]$$
  where $\left(x_{s},x_{e}\right)$ and ${(y}_{s},y_{e})$ are the pixel coordinates of the start and end points of the dimension; d is the dimension value; and λ is the direction indicator factor (0 for horizontal dimensions, 1 for vertical dimensions); $id_{x,s}$ and $id_{x,e}$ are the start and end node IDs matched in the X-direction; and $id_{y,s}$ and $id_{y,e}$ are the start and end node IDs matched in the Y-direction.
4. Coordinate Calculation Based on Dimension Chains and Proportions. After obtaining the association array R, a hybrid solution method of “dimension chain recurrence + proportional interpolation” is adopted to calculate the actual node coordinates. First, Base Establishment and Recurrence: The bottom-left node is set as the origin (0, 0). The association array R is traversed; if node i is known and connected to node j by dimension D, the coordinates of j are deduced. Additionally, Proportional Interpolation Solution: For special nodes not directly annotated by dimensions (such as the mid-point load position, as shown in Figure 5), utilizing the approximation principle of affine invariance in perspective projection, the actual physical coordinates are calculated via interpolation based on the proportional pixel distance between the point and adjacent known nodes on the image.
5. Element Connectivity Identification and Topology Construction. Upon completing the solution for actual node coordinates, the element connection relationships must be determined. All node pairs (i, j) are traversed, and a detection band with a width of 5 pixels is constructed along the line connecting the two nodes. The proportion of effective structural pixels within the band is calculated; if it exceeds a set threshold, the node pair is judged to be connected, constituting a structural element. Following the “Minimum Element Principle,” if three or more nodes are collinear, they are segmented into multiple continuous elements, ultimately generating an element connection list.

#### 3.4.2. Load Recognition and Numerical Association

In the solution system of the Matrix Displacement Method, the structural equilibrium equation $\left[K\right]\left\{\Delta \right\}=\left\{P\right\}$ serves as the core, where $\left[K\right]$ is the global stiffness matrix determined by the topology and element properties identified in Section 3.4.1, and $\left\{P\right\}$ is the combined nodal load vector determined by external loading conditions. Therefore, to construct a complete mechanical computational model, the problems of load recognition and quantification must be resolved.

Common load forms in plane frames include concentrated forces F, bending moments M, and distributed loads q. Given the significant differences in graphical features and mechanical mechanisms among these three load types, this section establishes targeted recognition and parsing workflows for each. The aim is to extract the three key elements—“magnitude, direction, and point of application”—and ultimately unify them into nodal load data readable by the program:

1. Recognition of Concentrated Forces and Nodal Association. Concentrated forces typically appear as straight arrows pointing towards nodes in drawings. First, the load detection model is used to detect and locate the overall region of the concentrated force, obtaining its center point $P_{1}(x_{1},y_{1})$. The load arrow detection model is then employed to detect arrow features and obtain the arrow center point $P_{2}(x_{2},y_{2})$. A direction vector $\upsilon =P_{2}-P_{1}$ pointing from P1 to P2 is constructed. By analyzing the signs of the components of υ on the X and Y axes, the direction of action of the concentrated force in the global coordinate system is determined, thereby establishing the sign of the load. Second, OCR character recognition is performed on the load annotation region. Combined with regular expression processing, the numerical magnitude is extracted, and the final sign of the concentrated force is determined based on the direction information. Finally, based on the nearest-neighbor principle, the arrow center point P2 is matched against the set of node pixel coordinates, and the node with the minimum distance is judged as the point of application for the concentrated force.
2. Recognition of Bending Moments and Nodal Association. Bending moments are usually represented by curved arrows, where the core challenge lies in determining the direction of rotation (clockwise/counter-clockwise). First, the load detection model locates the overall center point $P_{3}(x_{3},y_{3})$ of the bending moment. The load arrow detection model detects the bending moment arrow, and edge detection algorithms are used to extract the arrow’s contour. The intersection of the two main boundary lines is calculated as the arrow tip point $P_{4}(x_{4},y_{4})$, and the midpoint of the line connecting the tail endpoints of the boundary lines is taken as the arrow tail point $P_{5}(x_{5},y_{5})$. Based on the vector cross product principle to determine the rotation direction, vectors $V_{tip}=P_{4}-P_{3}$ and $V_{tail}=P_{5}-P_{3}$ are constructed to calculate the 2D vector cross product $\theta =V_{tip}\times V_{tail}$. The sign of θ determines whether the moment is counter-clockwise or clockwise, thereby establishing the sign of the moment value. The processes for numerical extraction and matching the point of application (spatial proximity principle) remain consistent with those for concentrated forces.
3. Recognition of Distributed Loads and Equivalent Conversion. Distributed loads appear as arrays of arrows distributed along structural members. The processing difficulty lies in the mechanical conversion from “element loads” to “nodal loads.” The direction of the distributed load can be determined by extracting just a single prominent arrow within the array; its numerical information is extracted via OCR technology and matched with load primitives using the spatial proximity principle. The objects acted upon by distributed loads are structural elements rather than nodes. In the Matrix Displacement Method, non-nodal loads cannot be directly incorporated into the nodal equilibrium equations. Therefore, by spatially matching the distributed load region with identified elements, the corresponding element is determined. Then, based on the element length, boundary conditions, and load magnitude identified by the program, the fixed-end reaction forces generated at both ends of the element are calculated. These forces are inverted in sign to be converted into equivalent nodal loads and superimposed onto the load array of the corresponding nodes, thus completing the final assembly of the computational model.

#### 3.4.3. Support Classification and Boundary Extraction

In the computational framework of the Matrix Displacement Method, the unconstrained global stiffness matrix is typically a singular matrix that possesses no inverse matrix, rendering the system of equations unsolvable. As the connection interface between the structure and external constraints, the essential role of a support is to apply constraint conditions to the nodal degrees of freedom (DOFs). This renders the displacements of specific nodes as known quantities, thereby providing solvability conditions for the stiffness equations. Therefore, accurately identifying support types and converting them into program-readable displacement boundary conditions constitutes the final critical step in constructing a complete mechanical model.

In plane frames, common support forms primarily include fixed supports, pinned supports (fixed hinged), sliding supports, and roller supports (movable). Significant differences exist in the displacement conditions corresponding to these different supports. To adapt to the computational program, this study adopts a coding method of “State Indicator Code + Known Displacement Value” (referring to the aforementioned fifth data group format):

1. State Indicator Code (α): Used to mark the unknown state of a DOF. α=1 indicates that the displacement in this direction is unknown and must be solved via equations; α=0 indicates that the displacement in this direction is known.
2. Known Displacement Value (u): When α=0, a specific forced displacement value is entered; when α=1 this value is invalid (defaulting to 0.0).

This study primarily targets general engineering scenarios and provisionally sets the displacement boundary conditions as homogeneous boundary conditions, implying that no forced settlement or rotation occurs at the supports (u=0). Based on this assumption, the mapping relationship of constraint information for the four typical support types is presented in Table 4.

### 3.5. Static Analysis Calculation Program

**Figure 6:**
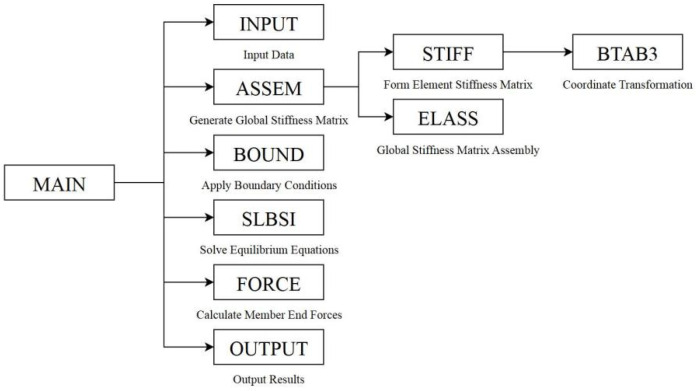
Static Analysis Program Flowchart.

New methods and protocols should be described in detail, while well-established methods can be briefly described and appropriately cite Following the completion of the conversion from visual features to structured data, the next step involves inputting this data in file format into the static analysis calculation program developed in this section, which is based on the Matrix Displacement Method. The program adopts a modular design approach, comprising six primary modules: the Data Input Module, the Global Stiffness Matrix Generation Module, the Boundary Condition Processing Module, the Equation Solving Module, the Member End Force Calculation Module, and the Result Output Module. Specifically, the Global Stiffness Matrix Generation Module encompasses element stiffness generation, coordinate transformation, and the assembly of element stiffness matrices into the global stiffness matrix. The invocation of subroutines by the main program and the interrelationships among subroutines are illustrated in Figure 6.

#### 3.5.1. Program Design and Algorithm Principles

The calculation module in this section adopts an Object-Oriented Programming (OOP) paradigm, encapsulating the FrameSolver class to achieve automated management of the entire structural analysis lifecycle. The core computational logic strictly follows the standard procedures of the Matrix Displacement Method in structural mechanics, establishing the structural stiffness equation in the global coordinate system:

(13) $$\left[K\right]\left\{\Delta \right\}=\left\{P\right\}$$

where $\left[K\right]$ is the global structural stiffness matrix, $\left\{\Delta \right\}$ is the nodal displacement vector, and $\left\{P\right\}$ is the nodal load vector. In consideration of the sparsity and symmetry of the stiffness matrix, the program is optimized in terms of storage and solution:

1. Half-Bandwidth Storage: Utilizing matrix symmetry, only the half-bandwidth elements of the global stiffness matrix are stored, effectively reducing memory usage.
2. Variable-Bandwidth Optimized Solver: A linear equation solver optimized for the storage structure is designed. Adopting an improved algorithm based on Gaussian elimination, elimination and back-substitution operations are performed only on elements within the effective bandwidth, thereby enhancing solution efficiency.

#### 3.5.2. Calculation Workflow

The execution workflow of the program primarily consists of the following six key modules:

1. Data Parsing and Pre-processing: The program reads the standardized structural data file generated in Section 3.4 and parses the topological relationships of the frame. It initializes the total number of nodes, total number of elements, and material properties (Elastic Modulus E, Moment of Inertia I, etc.), and dynamically allocates the memory space required for calculation based on these parameters.
2. Global Stiffness Matrix Assembly: Iterating through all elements, the program calculates the element stiffness matrix in the local coordinate system. It then utilizes the coordinate transformation matrix to convert it to the global coordinate system and superimposes it onto the global stiffness matrix according to node IDs.
3. Application of Boundary Conditions: Based on the support types, the “Set-to-1 method” is employed to modify the global stiffness matrix and load vector, thereby eliminating rigid body displacements.
4. Equation Solving: The optimized solver is invoked to solve the processed system of linear equations, obtaining the generalized displacement vectors of all nodes in the global coordinate system.
5. Member End Force Calculation: Utilizing the obtained nodal displacements, the member end internal forces of each element in the local coordinate system are calculated via back-substitution.
6. Result Output: The calculated nodal displacements, support reactions, and element internal forces are output in a structured format, serving as the data source for the subsequent automatic plotting of bending moment, shear force, and axial force diagrams.

### 3.6. Visualization of Internal Force Diagrams

Upon obtaining the member end internal forces and nodal displacements output by the static analysis program, this section aims to achieve the graphical representation of the calculation results. First, the program parses the results file from the static analysis to extract the axial force, shear force, and bending moment values for each element, and establishes a plotting coordinate system by combining the geometric topological information of the elements.

Addressing the distinct plotting requirements for bending moment, shear force, and axial force diagrams, the program maps the internal force values of each element into geometric offsets perpendicular to the member axis according to a set scale, in strict accordance with the standard sign conventions of structural mechanics. Through coordinate transformation, the algorithm calculates the plotting coordinates of element start/end points and key control points within the global coordinate system. It then utilizes the Matplotlib plotting library (version 3.7.2) to connect these points, forming closed envelope lines of the internal force distribution, and superimposes them onto the original frame structure diagram.

Furthermore, the program incorporates an automatic annotation feature capable of displaying specific internal force values at member ends and extremum points. This process ultimately generates clear and proportionally coordinated diagrams for the three internal forces, completing the transformation from abstract data to visual internal force diagrams.

## 4. Case Study and Validation

To verify the effectiveness and accuracy of the “Visual Recognition–Parameter Extraction–Mechanical Calculation” technical method, this study first constructed a test dataset comprising 130 plane frame images (covering standard textbook exercises and simplified engineering drawings). In large-scale testing, the overall success rate of this method in structural topological reconstruction and internal force solution exceeded 90%, verifying the robustness of the algorithm across different line styles and annotation specifications.

Based on this statistical validation, this chapter selects two cases for verification analysis: one is a typical exercise from structural mechanics teaching, and the other is a double-span portal frame structure common in engineering design. This workflow utilizes the trained detection model to perform primitive recognition and geometric regularization on the raw images, and automatically assembles the generated structured data into the static analysis program to complete the solution and plotting. Finally, through quantitative comparison with a standard solver, the relative error is analyzed and the total elapsed time of the entire workflow is recorded to verify the accuracy and efficiency of the proposed method.

### 4.1. Validation with Structural Mechanics Exercise

**Figure 7:**
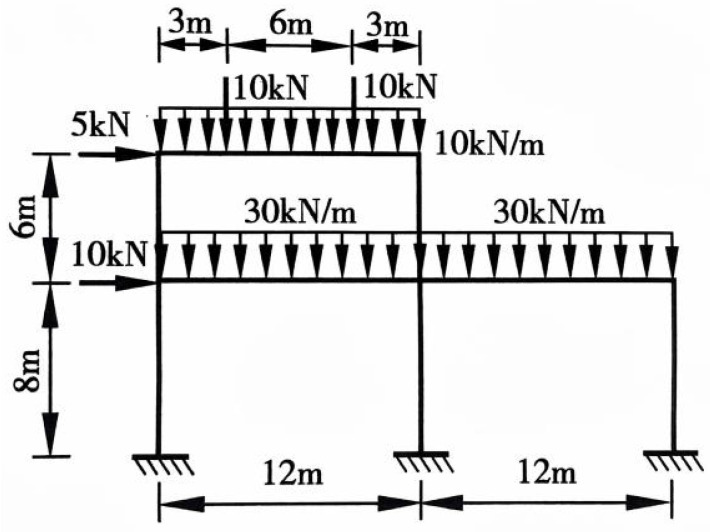
Structural Mechanics Exercise.

The selected structural mechanics exercise involves a two-span, two-story frame. The load distribution and structural dimensions are illustrated in Figure 7. The geometric and material properties are defined as follows: for all transverse beams, the cross-sectional area is A_1_ = 0.35 m^2^ and the moment of inertia is I_1_ = 0.0388 m^4^; for all columns, the cross-sectional area is A_2_ = 0.24 m^2^ and the moment of inertia is I_2_ = 0.0388 m^4^; the elastic modulus of the material is E = 3.0 × 10^4^ MPa.

**Figure 8:**
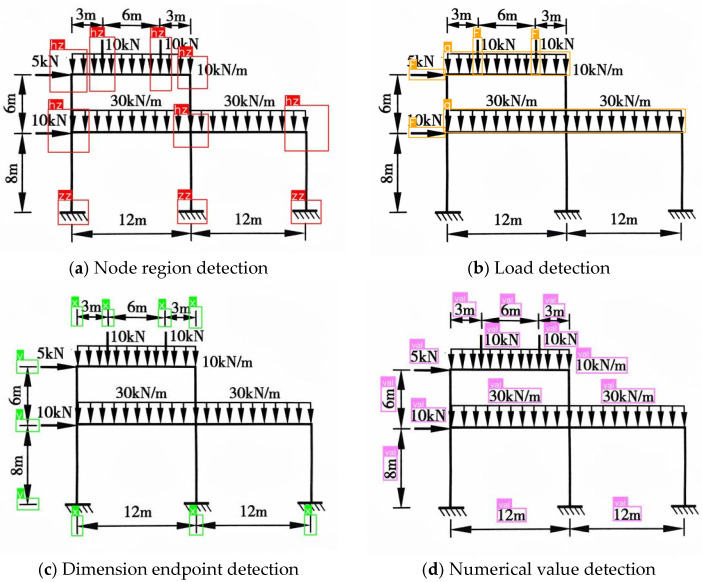
Global Object Detection of the Frame.

**Figure 9:**
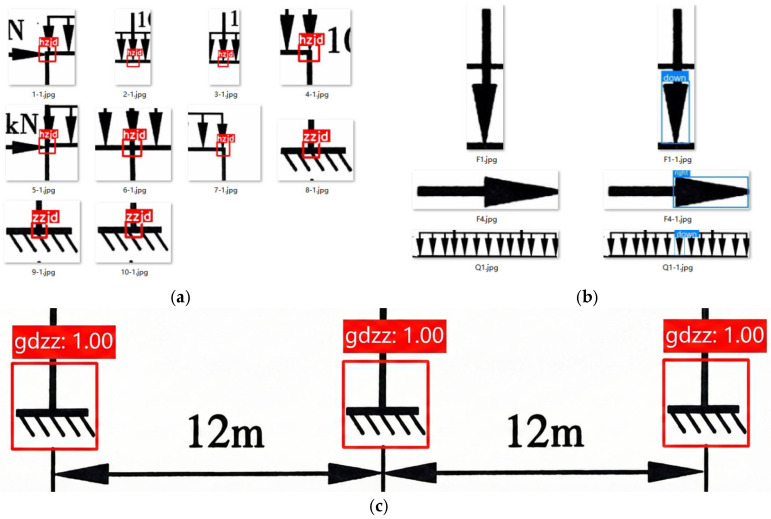
Local Refinement Detection and Support Classification. (**a**) Fine-grained node detection. (**b**) Load arrow detection. (**c**) Support classification. The label “gdzz” in (**c**) denotes the “fixed support” category in the support classification dataset.

The plane frame recognition process was conducted, and the intermediate results are as follows:

1. Global Object Detection: The YOLOv8 model successfully located all key elements within the frame. As shown in Figure 8, the model accurately detected 10 node regions, 4 concentrated loads, 2 distributed loads, and all dimension endpoints and numerical values, with no missed detections or false positives.

2. Local Refinement Detection and Support Classification (Figure 9): For the detected node regions (Figure 8a), cropping and secondary recognition were performed to output the pixel coordinates of the nodes. For the loads (Figure 8b), secondary detection was conducted to correctly judge the direction of action for both concentrated and distributed loads based on arrow direction. For support node regions, the classification model correctly identified them as “Fixed Supports,” determining the boundary conditions at the bottom of the frame to be fully constrained.

Based on the pixel information obtained from image recognition, the structured reconstruction method described in Section 3.4 was employed to transform the discrete visual information into data files directly readable by the static analysis program. To intuitively demonstrate this conversion result, Table 5 lists examples of various parts of this structured data file along with explanations of their corresponding mechanical meanings. This formatted output not only ensures machine readability of the data but also facilitates rapid manual verification of the recognition results.

**Figure 10:**
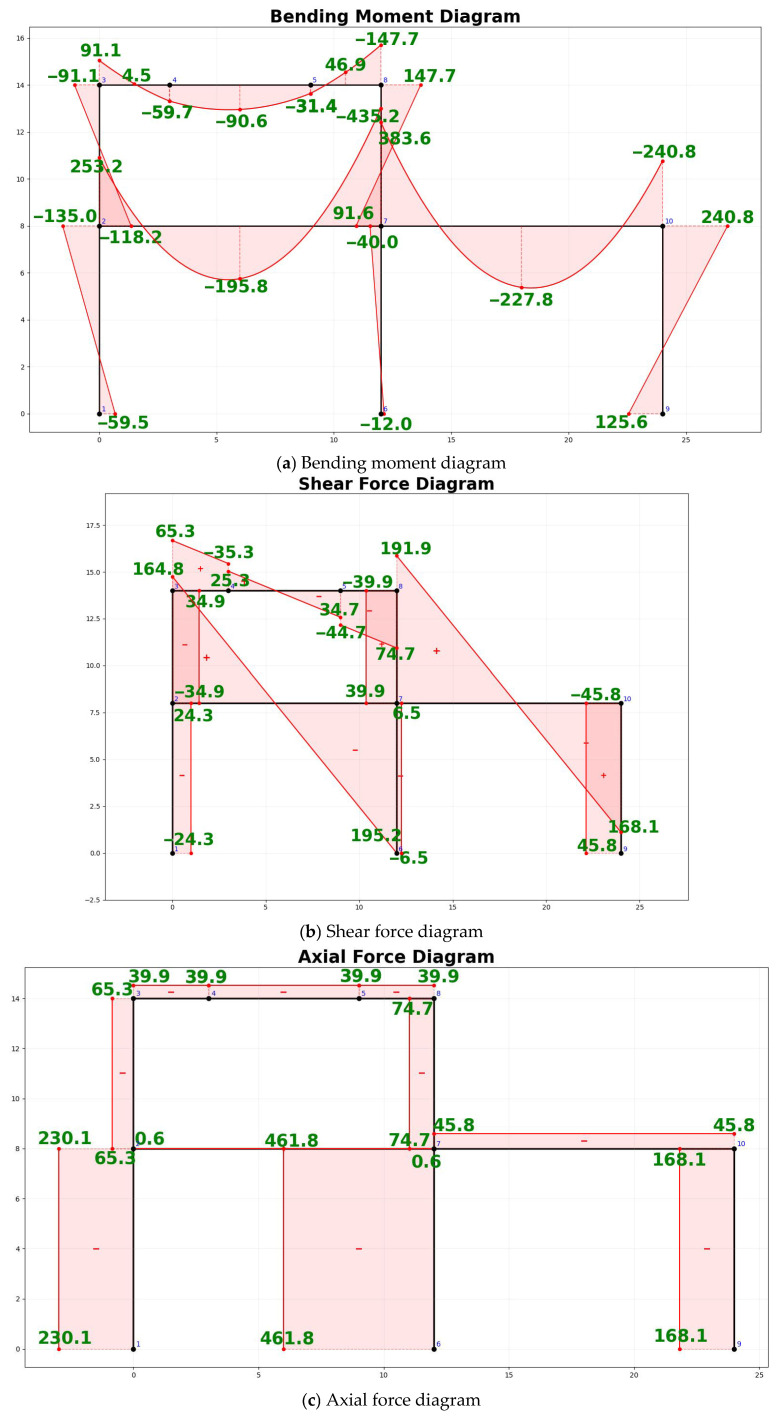
Internal force diagrams of the exercise frame. The blue numbers represent the node numbers of the frame, and the red symbols indicate the sign of the internal forces.

Subsequently, the frame data table was input into the static analysis module developed in Section 3.5. Based on the Matrix Displacement Method, the module automatically assembled the global stiffness matrix and performed the solution. For the two-span, two-story frame in this case, the program outputted nodal displacements, support reactions, and member end internal forces upon completion. Using the calculated discretized internal force values combined with the parametric plotting algorithm described in Section 3.6, the internal force diagrams of the frame were drawn (Figure 10). The bending moment diagram (Figure 10a) adheres to the structural mechanics convention of “drawing moments on the tension side.” As observed in the diagrams, elements subjected to distributed loads correctly superimposed quadratic parabolic shapes, with moment values annotated at both ends and the mid-span of the elements. Simultaneously, at rigid nodes, the moment values of the connected members achieved equilibrium. Furthermore, the shear force diagram (Figure 10b) clearly reflects the sudden changes (discontinuities) at the points of concentrated force application, while the axial force diagram (Figure 10c) accurately expresses the compression state of the columns.

Finally, to validate the correctness of the aforementioned results, a model was established for the same example using the “Structural Mechanics Solver” software for comparison. Numerically, the maximum nodal bending moment calculated in this case is consistent with the result from the “Structural Mechanics Solver.” Graphically, the internal force diagrams generated in this case perfectly align with the graphs output by the standard software in terms of trend, shape, and values at key points.

### 4.2. Analysis of Double-Span Portal Frame

**Figure 11:**
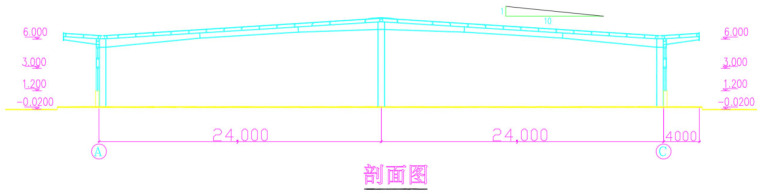
Transverse cross-section of a toy production workshop.

This case study utilizes the actual design drawings of a production workshop from a toy company as the background for engineering validation (Figure 11). The main structural system of the workshop is a double-span double-slope portal frame, with overall plan dimensions of 144 m × 48 m. The total transverse span is 48 m, composed of two 24 m spans. The column height is 6 m on the left and right sides, and 8.4 m for the center column, with a longitudinal column spacing of 8 m. Cantilevered canopies with a width of 4 m are provided on the exterior of Axis A and Axis C.

**Figure 12:**
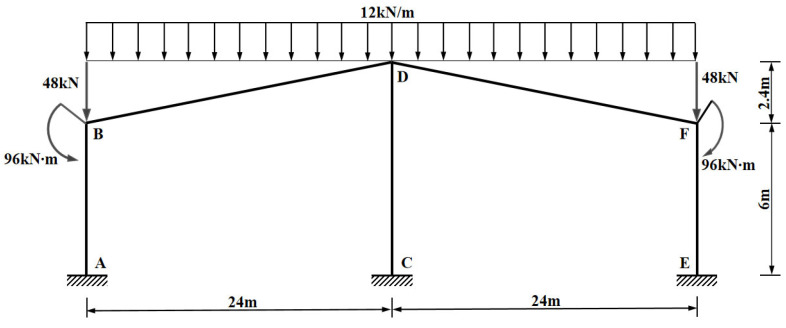
Simplified structural schematic of the production workshop.

To construct a standardized input image suitable for the visual recognition program proposed in this paper, while ensuring the mechanical accuracy of structural calculations, the original CAD engineering drawings were subjected to the following simplification and equivalence processing (as shown in Figure 12), in accordance with *the Code for Design of Steel Structures*, the *Load Code for the Design of Building Structures*, and fundamental principles of structural mechanics:

1. Geometric Simplification: The centerlines of beam and column members were extracted to construct a wireframe model, and the column base supports were uniformly simplified as fixed supports. In drawing the simplified structural schematic, the proportional relationship between geometric line segment lengths and annotated numerical values was intentionally dissociated. That is, the ratio between pixel lengths in the diagram does not correspond to the ratio between the dimension annotations. This ensures that even in cases of scale disproportion or deformation in the structural diagram, the calculation results remain precise provided the annotations are correct.
2. Load Simplification: The characteristic value of the dead load (including member self-weight) is 0.5 kN/m^2^ and the characteristic value of the roof live load (non-accessible) is 0.5 kN/m^2^. The frame column spacing is B = 8 m, and the canopy cantilever width is L_C_ = 4 m. Therefore, the design value of the area load ω is calculated as:
  (14) $$\omega =1.3\times 0.5+1.5\times 0.5=1.4\;kN/m^{2}$$
  The area load is converted into a line load:
  (15) q=ω×B=1.4×8=11.2 kN/m
  Rounding to an integer, q = 12 kN/m is applied vertically downward on the pitched beams. The canopy load is equivalently converted into a concentrated force P and a concentrated moment M:
  (16) $$P=q\times L_{C}=12\times 4=48\;kN$$
  (17) $$M=P\times \frac{L_{C}}{2}=48\times \frac{4}{2}=96\;kN\cdot m$$
  In the simplified structural schematic, a vertical concentrated force P = 48 kN and a corresponding nodal moment M = 96 kN∙m (counter-clockwise for the left column, clockwise for the right column) are applied to the column tops at Axis A and Axis C, respectively.

**Figure 13:**
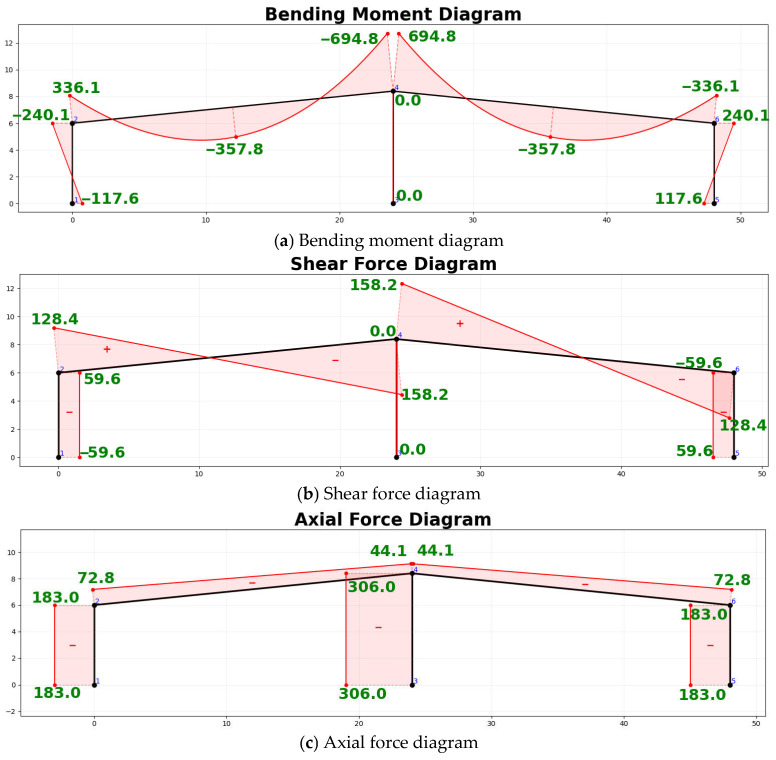
Internal force diagrams of the double-span portal frame. The blue numbers represent the node numbers of the frame, and the red symbols indicate the sign of the internal forces.

Overall process verification was conducted on the simplified portal frame schematic. The technical method proposed in this paper rapidly completed node localization, topological reconstruction, and static analysis. The generated internal force diagrams are shown in Figure 13.

Finally, the “Structural Mechanics Solver” was utilized to establish a model with identical parameters for comparative analysis. Graphically, the generated internal force diagrams are completely consistent with the output from the solver in terms of variation trends, with the correct superposition of distributed loads. Numerically, values at the nodes were selected for verification, and the calculated values in this paper are identical to the theoretical values.

### 4.3. Comparative Analysis of Comprehensive Efficiency and Performance

To objectively evaluate the application value of the proposed method in practical engineering and teaching scenarios, this section conducted full-workflow time statistics for the aforementioned typical cases. Traditional manual calculation and general-purpose Finite Element Analysis (FEA) software (SAP2000 v21) were introduced as baseline control groups to unfold a comparative analysis from the two dimensions of modeling efficiency and operational complexity. First, the calculation overhead of each stage of the proposed method from “image input” to “internal force diagram output” was statistically analyzed. As shown in Table 6, for the two standard cases, the system’s end-to-end processing time was controlled within 30 s. Notably, the inference time of the YOLOv8 model accounted for only about 15% of the total duration, with the primary time overhead concentrated in the parameter input and solution stages of the static analysis program. The time consumption for each stage is detailed in Table 6.

To intuitively demonstrate the efficiency advantages of the proposed method, the aforementioned results are compared with the standard operating times of traditional methods, as shown in Table 7.

1. Traditional Manual Solution: This relies on manual geometric modeling, derivation of equilibrium equations, and numerical calculation. For statically indeterminate structures such as the double-span portal frame, a skilled engineer or student typically requires 20–30 min to complete the full calculation and plotting, and the process is highly prone to calculation errors.
2. Commercial Software (SAP2000 v21): Although its numerical solution core requires only milliseconds, its “human–computer interaction cost” is extremely high. Users must undergo cumbersome pre-processing steps involving defining grids, drawing members, assigning cross-sections, applying loads, and setting boundaries. For non-batch simple frame problems, the entire modeling process for a skilled operator typically requires 10–15 min.

**Table 7:** Comparison of Efficiency and Characteristics among Three Structural Analysis Methods.

Method Type	Avg. Total Time	Operational Complexity	Main Bottleneck	Usage Scenarios
Traditional Manual Calculation	20–30 min	High (Error-prone)	Tedious matrix operations and equation derivation	Theoretical exams, learning basic principles
SAP2000 Modeling	10–15 min	Medium (High entry barrier)	Complex GUI interaction and parameter settings	Large-scale complex structures, detailed design
Proposed Method	20–30 s	Low (Automated)	Dependence on image clarity and shooting angle	Homework grading, rapid on-site verification

Comparative Conclusion: As demonstrated in Table 7, the automated method proposed in this paper reduces the time cost of structural analysis from the “minute-scale” to the “second-scale,” improving efficiency by approximately 30–40 times compared to manual modeling in SAP2000 v21. This method successfully eliminates the complex modeling pre-processing barrier of commercial software, achieving “What You See Is What You Get” rapid analysis, and effectively fills the application gap between manual calculation and large-scale software.

## 5. Discussion

The experimental results of this study have confirmed the feasibility of applying computer vision technology to the structural analysis of plane frames, successfully constructing a complete analysis workflow ranging from “high-precision primitive detection” to “automated internal force generation.” Based on the primitive-level dataset containing 7030 images, the YOLOv8 model achieved a Mean Average Precision (mAP@0.5) of 99.1% on the validation set. This high-precision perception foundation provides a reliable guarantee for subsequent topological reconstruction, effectively reducing the risk of initial topological errors caused by missed detections of primitives. Building on this, this section will focus on discussing how this method handles visual error propagation, the generalization performance of the method, and its current limitations.

### 5.1. Error Propagation Analysis and Robustness Mechanism

Addressing the inevitable pixel-level detection errors in computer vision, this study introduces a robust geometric regularization mechanism that effectively blocks the propagation of errors to the mechanical analysis stage. This mechanism is designed based on the spatial distribution characteristics of structural schematics and includes the following three key strategies:

1. Node Alignment: Statistical analysis indicates that the Euclidean distance between distinct nodes in standard problem sets typically exceeds 100 pixels, while the detection jitter deviation of collinear nodes is generally within 10 pixels. Therefore, this paper sets a tolerance threshold of 30 pixels to align nodes along horizontal or vertical axes. This threshold setting is sufficient to cover detection noise yet far smaller than the minimum physical spacing between nodes, thereby eliminating coordinate drift during the reconstruction phase.
2. Dimension Association: The mapping between dimension values and nodes follows the same spatial tolerance logic described above. By constructing a spatial adjacency matrix, the system ensures that dimensional parameters are accurately assigned to the corresponding geometric primitives, avoiding parameter matching errors caused by minor visual positional offsets.
3. Topology Verification: To ensure the accuracy of member connections, pixel density detection (with a threshold set at 80%) is introduced between node pairs. This strategy guarantees that member elements are generated only when high-confidence connecting lines exist, effectively filtering out false topological connections caused by visual false detections.

Through the above mechanisms, this method successfully bridges the “semantic gap” between image pixels and mechanical models, ensuring that the finally generated mechanical model is unaffected by minor displacements in primitive detection, thereby greatly enhancing the robustness of the algorithm.

### 5.2. Analysis of Generalization, Scalability, and Efficiency

To verify the generalization capability of the method, this study constructed a test dataset containing 130 frame exercises of different types. Test results indicate that the structured reconstruction algorithm achieved a solution success rate exceeding 90% when handling problems with different line styles and annotation specifications, demonstrating its stability in standard printed drawing scenarios.

Regarding scalability, although this study mainly focuses on plane frames, the proposed “primitive detection-topology reconstruction” methodology holds broad application potential. Its core logic—recovering connection relationships by identifying nodes and members and utilizing geometric constraints—is equally applicable to other skeletal structures such as Trusses or Grillages. Achieving transfer across structure types would only require extending the category definitions for object detection.

From the perspective of engineering efficiency, this method significantly optimizes existing workflows. Compared to the manual modeling process required by traditional finite element software (e.g., SAP2000 v21), which typically takes 10–15 min, this method reduces the entire process from “image input” to “internal force diagram output” to 20–30 s. This second-scale automated processing capability demonstrates significant application advantages in assisting structural mechanics teaching demonstrations and rapid on-site engineering verification.

### 5.3. Limitations and Future Improvement Directions

Despite the expected results achieved by the proposed method in the automated analysis of standard plane frames, certain deficiencies remain when dealing with complex mechanical loading conditions and non-ideal images, limited by the coverage of the current dataset and the simplification of the algorithmic logic. These issues need to be improved in future research:

1. The recognition accuracy for complex and dense load conditions needs improvement. The current detection model primarily targets single or sparsely distributed standard load forms. When confronting complex combined loads or high-density load distributions, severe pixel overlap among primitives renders the Non-Maximum Suppression (NMS) mechanism of the detection model susceptible to missed detections or false positives, leading to either missing or redundant load information in the mechanical model.
2. Absence of analysis capabilities for non-load factors. The current program logic strictly addresses static responses under the action of “external force loads.” For internal force changes induced by non-load factors—such as support displacement (prescribed displacement), temperature changes, or fabrication errors—the system is currently unable to calculate structural internal forces, as the present dataset does not encompass the relevant annotation symbols for these conditions.
3. Limitations in generalization for hand-drawn sketches and precision of text parameter extraction. The current detection model was trained primarily on printed samples. When applied to uncontrolled hand-drawn sketches featuring extremely scribbled lines, severe scale distortion, or numerous alteration traces, the robustness of topological relationship recognition requires improvement. Furthermore, the extraction of load values and geometric dimension text currently relies on general-purpose OCR engines. To meet the high-precision requirements of engineering verification, future research needs to further integrate specialized OCR technologies optimized for engineering symbols and combine them with the association algorithms proposed in this paper to realize precise matching between numerical values and components.

## 6. Conclusions

This paper proposes an automatic static analysis method for plane frame structures based on computer vision. By integrating deep learning object detection technology with the theory of the Matrix Displacement Method, it achieves the automated processing workflow from “capturing structural schematics via photography” to “generating internal force diagrams.” The main conclusions are as follows:

1. Validated the effectiveness of the YOLOv8-based recognition method for plane frame structural primitives. Addressing the issue that existing research lacks recognition of mechanical model components, a multi-category dataset comprising 7030 images was constructed. Experimental results indicate that this method effectively identifies structural primitives on printed plane frame schematics, providing precise data input for structural calculations.
2. Proposed a structured reconstruction method from visual semantics to mechanical models. To bridge the “semantic gap” between computer vision data and structural mechanics computational data, a structured data conversion algorithm was designed. This algorithm successfully transforms image recognition results into structured data required by the Matrix Displacement Method, including nodal encoding, load inputs, and boundary conditions.
3. Constructed a novel rapid analysis method for plane frames based on visual perception. This paper combines deep learning algorithms with a traditional Matrix Displacement Method program to realize the automation of structural analysis. Experimental validation demonstrates that this method significantly enhances the efficiency of structural mechanics teaching demonstrations and rapid engineering estimation, offering a new technical pathway for intelligent structural analysis.

## Figures and Tables

**Table 1:** Details of Datasets.

Stage	Dataset	Target Classes	Sample Size	Training Purpose
1	Node Region Dataset	Load nodes, Support nodes, Normal nodes	670	To locate the approximate range of nodes and prepare for ROI cropping.
2	Fine-grained Node Dataset	Nodes	1570	To precisely locate node coordinates within the ROI for establishing the stiffness matrix.
3	Support Classification Dataset	Fixed supports, Pinned supports, Sliding supports, Roller supports	1710	To determine the types of boundary conditions.
4	Dimension Symbol Dataset	X/Y-direction dimension limit endpoints	670	To extract actual geometric dimensions.
5	Load Dataset	Concentrated force (F), Bending moment (M), Distributed load (q)	670	To identify the types of load actions.
6	Load Vector Dataset	Force arrows, Moment arrows	1280	To determine the direction of load actions.
7	Value Localization Dataset	Load values, Dimension values	460	To locate regions for OCR recognition.

**Table 2:** Training Parameters of Models.

Model Name	Input Size	Epochs	Batch Size	Optimizer	Initial Learning Rate
Node_area	1024	50	4	SGD	0.01
Dimension	1024	50	4	SGD	0.01
Load	1024	50	4	SGD	0.01
Value	1024	50	4	SGD	0.01
Support_Cls	320	50	16	SGD	0.01
Node	640	50	8	SGD	0.01
Load Vector	640	50	8	SGD	0.01

**Table 3:** Model Performance Evaluation.

Model	Precision	Recall	mAP@0.5 (Top-1 Accuracy)	Inference Time (ms)
Node_area	0.997	0.993	0.995	28.83
Dimension	0.998	0.998	0.995	28.37
Load	0.997	0.997	0.995	28.55
Value	0.975	1.000	0.987	19.15
Support_Cls	/	/	0.972 (Acc)	4.05
Node	0.979	0.984	0.985	10.76
Load Vector	1.000	1.000	0.995	13.84

**Table 4:** Common Support Types and Boundary Constraint Information.

Support Type	$\alpha_{x}$	$\alpha_{y}$	$\alpha_{\theta }$	$u_{x}$	$u_{y}$	θ
Fixed Support	0	0	0	0.0	0.0	0.0
Pinned Support	0	0	1	0.0	0.0	0.0
Sliding Support	0	1	0	0.0	0.0	0.0
Roller Support	1	0	1	0.0	0.0	0.0

Where $\alpha_{x}$, $\alpha_{y}$, and $\alpha_{\theta }$ are the displacement state indicators, and $u_{x}$, $u_{y}$, and θ are the displacements in the corresponding directions.

**Table 5:** Structured data file example for mechanical analysis.

Group	Data Example (Standardized Format)	Description
I. Global Parameters	10, 10, 7, 3, 3 × 10^7^	Total Nodes, Elements, Loaded Nodes, Supports, E
II. Node Geometry	1, 0.0, 0.0	Node ID, X-coord, Y-coord
	2, 0.0, 8.0	
	……	
III. Element Connection	1, 1, 2, 2.4 × 10^−1^, 3.88 × 10^−2^	Element ID, Start Node, End Node, Area, Inertia
	2, 2, 3, 2.4 × 10^−1^, 3.88 × 10^−2^	
	……	
IV. Load Vector	2, 10, −180, −360	Node ID, F_X_, F_Y_, M
	3, 5, −15, −7.5	
	……	
V. Boundary Condition	1, 0, 0, 0, 0.0, 0.0, 0.0	Support Node ID, Constraints (u, v, θ), Prescribed Values
	6, 0, 0, 0, 0.0, 0.0, 0.0	
	9, 0, 0, 0, 0.0, 0.0, 0.0	

**Table 6:** Statistics of Workflow Time Consumption.

Stage	Time (Case 1)	Time (Case 2)	Remarks
Image Pre-processing	5 s	5 s	Includes manual ROI selection
YOLO Recognition	3.02 s	3.68 s	Model inference speed
Program Calculation	15 s	15 s	Includes material parameter input
Plotting Output	0.5 s	0.5 s	/
Total	23.52 s	24.18 s	

## Data Availability

The data that support the findings of this study are available from the corresponding author upon reasonable request.
